# Assessment of the Quality of Antenatal Care Services Provided by Health Workers Using a Mobile Phone Decision Support Application in Northern Nigeria: A Pre/Post-Intervention Study

**DOI:** 10.1371/journal.pone.0123940

**Published:** 2015-05-05

**Authors:** Marion McNabb, Emeka Chukwu, Oluwayemisi Ojo, Navendu Shekhar, Christopher J. Gill, Habeeb Salami, Farouk Jega

**Affiliations:** 1 Pathfinder International, Watertown, Massachusetts, United States of America; 2 Pathfinder International Nigeria, Abuja, Nigeria; 3 Boston University School of Public Health, Boston, Massachusetts, United States of America; University of Groningen, University Medical Center Groningen, NETHERLANDS

## Abstract

**Background:**

Given the shortage of skilled healthcare providers in Nigeria, frontline community health extension workers (CHEWs) are commonly tasked with providing maternal and child health services at primary health centers. In 2012, we introduced a mobile case management and decision support application in twenty primary health centers in northern Nigeria, and conducted a pre-test/post-test study to assess whether the introduction of the app had an effect on the quality of antenatal care services provided by this lower-level cadre.

**Methods:**

Using the CommCare mobile platform, the app dynamically guides CHEWs through antenatal care protocols and collects client data in real time. Thirteen health education audio clips are also embedded in the app for improving and standardizing client counseling. To detect changes in quality, we developed an evidence-based quality score consisting of 25 indicators, and conducted a total of 266 client exit interviews. We analyzed baseline and endline data to assess changes in the overall quality score as well as changes in the provision of key elements of antenatal care.

**Results:**

Overall, the quality score increased from 13.3 at baseline to 17.2 at endline (p<0.0001), out of a total possible score of 25, with the most significant improvements related to health counseling, technical services provided, and quality of health education.

**Conclusion:**

These study results suggest that the introduction of a low-cost mobile case management and decision support application can spur behavior change and improve the quality of services provided by a lower level cadre of healthcare workers. Future research should employ a more rigorous experimental design to explore potential longer-term effects on client health outcomes.

## Introduction

Nigeria is the most populous country in Africa, with over 174 million inhabitants, 44% of whom are below the age of 15 years. [1] Nigeria has a high average total fertility rate of 5.7 children per woman, with northern regions having higher fertility rates than southern Nigeria. [2] The uptake of antenatal care (ANC) is low in Nigeria; 37% of pregnant women did not attend ANC at all at the time of the 2008 Demographic and Health Survey, and among those who did access ANC, women had a median time of five months of pregnancy before their first visit, and less than half (44%) attend the recommended four or more ANC visits. [2]

According to the most recent estimates, Nigeria’s maternal mortality ratio is the tenth highest in the world, with an estimated 630 maternal deaths per 100,000 live births, and 40,000 maternal deaths annually. [3] There is now a critical need to support Nigeria’s efforts to make significant progress in achieving the Millennium Development Goal 5 target of reducing maternal mortality by 2015 through the government’s recently launched Saving One Million Lives (SOML) initiative. [4] With a dearth of skilled personnel at the primary health care level, Community Health Extension Workers (CHEWs) have increasingly filled the gap at primary health centers (PHCs). CHEWs have not received adequate technical support and most are unable to comprehensively address complications among high-risk women who present at the facility. [5] The low skill level of CHEWs and lack of adequate supervision adversely affects the quality of ANC. In larger PHCs, other health care workers (HCWs) including nurses and midwives, provide ANC services, in addition to the CHEWs.

In the last decade, mobile phone ownership and use has grown significantly in sub-Saharan Africa. With this growth has come the emergence of programs globally that use mobile technology to support health programs, otherwise known as mHealth. Mobile applications providing decision support and facilitating case management for health workers are one common application of mHealth programming. [6]

Studies in Africa and Asia have shown that mobile phone decision support applications for community health workers (CHWs) are acceptable and feasible and can improve clinician adherence to treatment protocols and national guidelines. [7–9] CommCare, an open source mobile phone decision support and case management platform developed by Dimagi Inc., allows users to develop custom applications that run on Java-enabled or Android-based phones or tablets. CommCare applications, or “apps,” are currently being implemented by over 50 organizations across 30 countries, mostly supporting CHWs in Asia and Africa, for a variety of health and development focus areas. [10] Research has shown that CommCare apps can improve the delivery of health care services and counseling, and increase the accountability of CHWs. [11],[12] One study found that CHWs are highly motivated to use mobile phones in their daily activities and that the use of the device engenders respect and social prestige in their communities. [13] CommCare applications supporting HCWs to adhere to integrated management of childhood illness protocols has shown reduction in clinical decision errors on average by 33% and increased protocol compliance by 30%. [14]

To our knowledge, no study has been conducted in Nigeria assessing the effect of implementing mobile phone decision support applications on the quality of ANC services offered by community health workers. In this paper, we present data regarding the effect of an mHealth decision support application used by CHEWs and other HCWs in 10 PHCs in Nigeria. We assessed two main outcomes: 1) the quality of services rendered, as reported by their clients and measured by 25 technical and counseling indicators; and 2) the level of satisfaction with ANC services received as reported by clients.

## Methods

### The m4Change ANC Application

The m4Change project was designed to support the Nigerian government’s SOML initiative that promotes the use of mobile technology to support implementation of maternal and child health programs. [4] The m4Change project was implemented by Pathfinder International from December 2012 to December 2013. Pathfinder developed a mobile ANC app using the CommCare mobile platform. The app was developed to support CHEWs/HCWs to provide higher-quality ANC services in 10 PHCs in Abuja federal capital territory and Nasarawa state in northern Nigeria.

CommCare allows users to develop mobile phone-based decision support and data collection applications that can be used on low-end Nokia feature phones or on the Android platform. The ANC decision support app developed by Pathfinder allowed CHEWs/HCWs to register and track individual clients over time using the app’s case management functionality. CHEWs/HCWs could automatically retrieve client records on the phone, displaying services received in previous visits and dynamically prompting the CHEW/HCW to provide services based on decisions and services offered in previous visits. Client records included demographic data, medical history, diagnostic information (e.g., blood pressure, height, weight, immunization status), and laboratory test results. The app contained decision support algorithms covering main areas of patient ANC services including screening for pre-eclampsia and management of obstetric danger signs. In cases where the HCW inputs test results (e.g., blood pressure, hemoglobin levels, proteins and glucose levels in urine, HIV status) that were out of normal range, the app generates a recommendation for a course of treatment, referral, and recommendations for tailored client follow-up. Table 1 describes the technical elements and protocols included in the app.

**Table 1:** Detail of Content of the m4Change ANC Application.

Application Module	Module Purpose	Data Elements
Client Registration	Register new ANC clients	Collects demographic information, past medical history, height, weight, blood pressure, and 1^st^ tetanus toxoid immunizations; calculates expected delivery date based on last menstrual period
Client Follow-up	The follow-up module is used when a client comes in for a subsequent visit that allows the user to view relevant information from registration or previous visits and input new clinical information	Including weight, blood pressure, and tetanus toxoid 2^nd^ or 3^rd^ immunizations
Lab/Examination	Prompts the HCW to record information during the examination or lab results	Maternal and fetal danger signs, tuberculosis, lab diagnostics (protein or glucose in urine, HIV test result, hemoglobin levels, syphilis, hepatitis B, malaria test, urinary tract and sexually transmitted infection test results). Fetal heart rate and lie are also captured in this module.
Health Counseling	Allows HCWs to play 11 different audio-recording clips	Nutrition, iron/folic acid, hygiene, birth planning, ANC visits, malaria, immunizations, dangers signs, postpartum contraception, HIV, and newborn care

The app also included a series of audio-recorded health counseling messages that were played for clients during ANC visits. Health counseling recordings included topics such as nutrition during pregnancy, use of iron/folic acid, maintaining hygienic practices, birth planning, number and timing of ANC visits, prevention against malaria, information about immunizations, danger signs during pregnancy, postpartum contraception, HIV, and newborn care. Counseling messages were adapted to the Nigerian context from standardized client education content developed by the Mobile Alliance for Maternal Action (MAMA) and were translated into two local languages, Pidgin English and Hausa. Health facilities with lower client loads conducted a one-on-one targeted health education session using the mobile phone speakers. In health facilities with more than 10 clients per day, the audio recordings were played through radios during group education sessions, as the volume on the mobile device was not powerful enough for large groups of people to hear clearly.

When a module was completed in the app, the data was automatically transmitted through the mobile network to be stored on a cloud server and aggregated on CommCareHQ. If CHEWs/HCWs were out of cellular network coverage, they were still able to use the application and store data locally until they entered an area with network coverage, when the phone could be synched.

In December 2012, 152 CHEWs/HCWs and 20 supervisors in 10 PHCs in Abuja federal capital territory and Nasarawa State were trained to use the mobile app. Each PHC was given either a Nokia phone or a tablet depending on the client load of the facility. Sites with a higher client load received a tablet for increased memory space to accommodate more client records. All devices were password protected to keep client information private and stored in the facility as HCWs were not allowed to use them for personal use. HCWs were trained to use the application with every ANC client they encountered at the facility during ANC days. During the project phase, HCWs still were required to fill in paper health management information system forms as mandated by the government.

### Study Design

The Health Research Ethical Committee (HREC) at the National Hospital, Abuja Nigeria reviewed and approved the protocol for this study. Participants gave verbal consent to participate in the study. No names or other identifying information was collected during the interviews. Written consent was not obtained due to low literacy levels of the women interviewed. The participant consent was recorded on the interview guide and signed by the data collector as receiving client consent. The study protocol was approved by HREC under the assigned number: NHA/EC/198/2012. The Ethical committee approved all verbal consenting processes for this study.

This was a pre/post-intervention study of first-time ANC clients above the age of 18 years at 10 PHCs in Abuja and Nasarawa, Nigeria. The main objectives of the study were to determine if introducing the mobile app: 1) improved the quality of ANC services provided, and 2) improved client satisfaction with ANC services provided. A quality score consisting of technical and counseling elements was developed and effectiveness was assessed by comparing scores from client exit interviews collected at baseline and one year after the intervention at endline.

The study was powered to detect a 15 percentage point improvement in individual quality attributes of ANC, from baseline to endline resulting in a target sample size of 267 client exit interviews. At the first level of selection, a total of 10 sites were randomly selected from the list of 20 m4Change project sites using the “randbetween” functionality in Microsoft Excel. A target client interview number was developed for each site based on client load on appointed ANC days. The target number was calculated using probability proportionate to size to reflect the diversity of client load at these sites.

### Data Collection

A baseline and endline client exit interview tool was adapted from the Measure DHS tool used for service provision assessment. Client exit interviews collected information about the clinical examination, health education and counseling received, as well as clients’ overall satisfaction with the care received during their first ANC appointment. The tools were translated into Hausa, the local language in the project states, and in English. The questionnaire was pilot tested in one PHC outside of the 10 study PHCs.

Twenty-five data collectors were trained to administer the exit interviews at baseline and endline. Interviewers visited sites for and conducted interviews on as many ANC days as was necessary to reach the individual site target. All women above the age of 18 (regardless of parity) who came for their first ANC visit were given the chance to participate in the interview. After verbal consent was obtained by the data collectors to participate in the study, exit interviews were conducted immediately after the client finished their first ANC visit in a private location in the PHC compound. Baseline interviews were conducted from November to December 2012. Endline interviews were conducted one year later (November to December 2013).

### Quality Score

Based on other similar frameworks for measuring ANC quality at primary health centers in the African context found in the peer reviewed literature, [15,16] we designed a quality score that aimed to classify two main domains related to ANC service delivery: technical care services and client health counseling. These domains were selected because the central instrument of the m4Change program was a mobile app deigned to help HCWs adhere to clinical protocols, improve client care offered, and standardize health education. The quality score consisted of 25 key elements, 12 of which relate to the technical care services component of the ANC and 13 related to the health counseling component of ANC. The app provided decision support functionality for all 25 quality attributes. The quality score was not weighted due to the need to test and validate weights assigned for individual elements. We were unable to find a validated, weighted quality score in the literature that was appropriate for our intervention. Developing and validating a weighted ANC quality score was beyond the scope of this study. For this quality score, it was a summation of elements that were reported by clients at exit from the facility as delivered or actions taken by health workers during the client visit. For example, clients were asked if their blood pressure or weight was taken. If yes, one point was added to the total score. Each element of ANC care, technical and counseling, was worth one point. The points received at baseline and endline were summed and endline changes were compared to baseline. We recognize that other domains—such as process, structural, and cost factors—also relate to the quality of health services. However, the m4change project and mobile application did not cover these aspects of care.

### Data Analysis

All data analysis was done using SPSS (IBM SPSS Statistics 20). Descriptive statistics were generated. Statistical significance was determined at the .05 level. The primary outcome measure was the increase in average of the composite quality score. Independent Sample T-Test was used to assess the difference in average ANC quality scores before and after implementation of the m4change project. The Chi-Square test was used to understand the differences in individual attributes before and after implementation of the m4Change project.

Monthly program monitoring reports were compiled and summarized to facilitate the compilation of lessons learned. Infrastructure assessments conducted at baseline and endline were compared and summarized to understand the availability of key commodities and equipment essential to deliver ANC services.

## Results

The m4Change project was implemented from December 2012 to December 2013 in 10 PHCs. Table 2 describes the demographic characteristics of the study population. A total of 266 ANC clients were interviewed directly after their first ANC visit for that pregnancy at baseline and endline in the 10 study PHCs. One client exit interview was removed from the analysis due to poor data quality. The average age for clients interviewed at baseline and endline was 24 years, and a little over half the clients were literate.

**Table 2:** Socio-demographic Variables for Client Exit Interviews.

Characteristic		Baseline (n = 267)	Endline (n = 266)
		% (n)	% (n)
Age years (sd)	Mean	24 (5.10)	24 (5.10)
Literacy (can read and/or write)	% (n)	56.7 (92)	46 (77)
Education level	Primary	35.2 (94)	19.6 (52)
	Post-primary/ Vocational	5.2 (14)	7.9 (21)
	Secondary/A-level	25.5 (68)	29 (77)
	College	7.1 (19)	6.4 (17)
	University	1.5 (4)	2.3 (6)
	No Schooling	25.5 (68)	34.8 (100)

Table 3 reports the mean scores of key ANC service delivery statistics at baseline and after the intervention, and is summarized using a quality score consisting of 25 key service delivery elements. The quality score is further divided into two main areas: technical care services and client health counseling. Overall, the quality score increased from 13.33 at baseline to 17.15 at endline (p<0.0001), with the most significant improvements related to health counseling.

**Table 3:** m4Change Quality Score Analysis from 10 PHC Study Sites.

Attributes as assessed by client report		Baseline (n = 267)	Post-m4change (n = 266)	P-value
***Technical Attributes Performed***		*% (n)*	*% (n)*	
Diagnostic approach	Woman informed of the due date of the baby	14.4 (38)	29.1 (77)	<.0001*
	Blood sample taken	93.1 (246)	96.9 (258)	0.042*
	Urine sample taken for Hgb, syphilis, blood type/RH and protein and WBCs	88.6 (235)	89.8 (239)	0.663
	HIV test performed	67.5 (175)	82.2 (209)	<.0001*
	Blood pressure measured	87.1 (230)	96.9 (258)	<.0001*
Provision of prophylactic drugs	Iron and folic acid tablets given	92.1 (244)	95.8 (254)	0.068
	Preventive malaria prophylaxis given	56.3 (146)	59.1 (152)	0.524
	Tetanus toxoid injection given	74.1 (195)	57.5 (152)	<.0001*
Physical exam	Fetal heart rate monitored	43.6 (116)	64.2 (171)	<.0001*
	Checked for swollen hands and face	30.5 (81)	41.4 (109)	0.009*
	Weight monitored	92.3 (243)	95.1 (253)	0.196
	Assessed for foul-smelling discharge	47.3 (126)	42.4 (112)	0.253
		*Mean (sd);*	*Mean (sd);*	
Quality score	**Sum of technical attributes performed score (total = 12)**	**7.77 (1.96)**	**8.44 (2.05)**	<.0001*
***Counseling Attributes Performed***		*% (n)*	*% (n)*	
General health education	Asked about previous pregnancies and complications	83.8 (218)	88.5 (232)	.119
	Explained potential danger signs	27.6 (72)	50.4 (132)	<.0001*
	Counseled on delivering with a skilled birth attendant	20.0 (52)	49.2 (129)	<.0001*
	Counseled on delivering at a health facility	39.1 (101)	74.9 (197)	<.0001*
	Counseled on the importance of exclusive breastfeeding	27.1 (70)	74.6 (197)	<.0001*
	Counseled on when to start breastfeeding	17.4 (44)	59.2 (147)	<.0001*
	Counseled on maternal and infant nutrition	31.2 (79)	76.1 (197)	<.0001*
	Counseled on postpartum contraceptive methods	20.7 (53)	64.3 (164)	<.0001*
Counseled on preventive measures	Given advice to use an insecticide treated net to prevent malaria	45.6 (119)	60.9 (162)	<.0001*
	Explained purpose of taking iron	29.8 (79)	70.2 (186)	<.0001*
	Explained side effects of taking iron	29.8 (78)	41.2 (109)	<.006*
	Explained malaria prevention, role of drugs and their side effects	20.4 (52)	31.7 (81)	<.004*
	Provider asked if client had any other concerns	56.5 (146)	72.2 (190)	<.0001*
		***mean (sd)***	***Mean (sd)***	
Quality score	**Sum of counseling attributes (Total = 13)**	**3.49 (5.45)**	**3.89 (8.67)**	<.0001*
		*Mean (sd);*	*Mean (sd);*	
TOTAL COMPOSITE QUALITY SCORE (Sum of technical and counseling attributes performed, out of a total of 25)		**13.33 (4.69)**	**17.15 (5.19)**	<.0001*

*Statistically significant at 0.05 level

In terms of technical attributes, the quality score increased from 7.77 at baseline to 8.44 at endline (p<0.0001) out of a maximum of 12. Half of the 12 elements in the technical domain showed a significant positive change from baseline to endline. The remaining five elements did not show a significant change, and one showed significant decline (tetanus toxoid injections). Among the six technical elements that did show significant improvement, performance in provision of HIV tests is most noteworthy, increasing from 67.5% to 82.2%. Measuring clients’ blood pressure increased from 87% to near universal at 97%.

Among the five technical elements that did not show a significant change, three (urine test, weight monitoring, and iron/folic acid provision) were already high at the baseline, 88% and above, and therefore had less margin to improve. These elements also require other inputs including ensuring a clear supply chain to ensure that iron/folic acid is available, urine tests are available and weight scales are function. The app and project did not address the overall supply chain or provide the commodities to the sites. The other two attributes (informed about the due date and assessed for foul smelling discharge) are areas for improvement; their baselines levels were low and they did not show a significant increase. Provision of tetanus toxoid injection declined significantly from 74% to 58%. This decline was largely due to the unavailability of the injection/commodities in some of the PHCs. It is worth noting that the m4Change project only introduced a mobile application and did not provide equipment or supplies to the PHCs. The improvement in many attributes without changing the commodities supply suggests that improvements can be made by simply reminding health workers of the required steps through the app.

The domain of health education had the highest quality score increase from 5.45 to 8.67 points (p<0.001). Twelve of the thirteen counseling elements showed statistically significant improvements from baseline to endline. At baseline, HCWs gave unstructured health talks prior to seeing individual clients for ANC days. During the intervention, structured audio recordings were played for clients on a variety of topics and HCWs used these clips during individual and group counseling sessions. At endline, more clients reported receiving counseling on key health topics including delivering with a skilled birth attendant, postpartum contraception and counseling on using insecticide-treated nets.

Table 4 depicts client satisfaction with the services they received during their ANC visit. Overall client satisfaction, as measured by proportion of clients who said they were very satisfied with the ANC services, increased from 75% at baseline to 83% at endline (p<0.05). Other aspects of client satisfaction relating to usefulness of information received, respectful treatment by provider, and client’s intention to return to the facility were already high at baseline (more than 91%) and did not significantly increase at the end of the intervention, but continued to remain high.

**Table 4:** Client Satisfaction during first ANC visit.

Client Satisfaction Indicator	Baseline % (n)N = 267	Endline % (n)N = 266	P Value
Client was very satisfied with the ANC services offered	75.4 (200)	83.3 (220)	0.025*
Information received during the visit was useful	91.1 (234)	93.8 (242)	0.239
Provider was respectful during the visit	98.8 (260)	99.2 (263)	0.647
Client will return for a follow-up visit	99.5 (246)	99.2 (243)	0.558

*Statistically significant at 0.05 level

## Discussion

In this study, there were two main objectives to assess the impact of the quality of services offered as a result of the intervention as well as the client satisfaction with services offered using the application. Through this study, we demonstrated that the m4Change intervention was in fact associated with higher quality of ANC scores, with these improvements observed in multiple domains of care. We also demonstrate a significant improvement in overall client satisfaction. The intervention seemed to have a particularly strong effect on counseling provided by the CHEWs. In addition, we also observed improved quality scores related to the frequency at which CHEWs performed more technical aspects of care, such as measuring blood pressure, obtaining blood or urine samples, and certain physical examination screenings for signs of pre-eclampsia. We hypothesize that these improvements were due to the elements of the app that guided CHEWs through the steps of a patient encounter.

The greatest improvements in the quality of services from baseline to endline were in the areas of client education and counseling. Health counseling during ANC has been shown to improve healthy behaviors leading to better health outcomes. [17] We also observed a significant increase in clients reporting having received health counseling for maternal health issues. Clients reported receiving counseling about birth preparedness and potential danger signs increased, which can have a positive impact on behavior change and awareness of the need for skilled delivery. Introducing audio-recorded counseling messages is a low-cost strategy for ensuring clients are exposed to standardized health education related to key issues surrounding pregnancy and childbirth. Standardized health counseling messages were introduced through this intervention and have the potential for affecting positive change in health behaviors that can affect maternal and child health outcomes. A recent review article assessing the literature on the impact of mHealth interventions shows studies have proven increase compliance to clinical protocols as a result of the intervention. However, no studies were reported to have shown in increase in the overall quality of health services offered as a result of introducing a mobile decision support job aid. [18]

In terms of the second objective of the study, to assess client satisfaction with services received, there was a significant improvement in overall satisfaction from baseline to endline. Clients reported satisfaction in quality of services provided during the visit was found to be statistically significant.

### Advantages

The study has many advantages in contributing to the growing field of evidence surrounding mHealth interventions in low and middle income countries. First, the study assessed a project that was implemented in real conditions of health facility operations in Nigeria, not based on assumptions or modeling. No other program inputs to the supply chain, additional training of health workers or support for clients to attend services were implemented in the project, highlighting the effect the mHealth application alone had on the quality of services. Additionally, no study, according to the authors knowledge, has been conducted to map the effects of a provider job aid on the overall quality of ANC services and client satisfaction in Nigeria. Therefore this study aimed to improve the literature base, in particular relation to the Nigerian context.

### Limitations

It is important to note that this study does not assess impact on health outcome, such as reductions in maternal mortality. This paper presents data from a demonstration project looking at the effect of introducing a mobile application on the quality of ANC service provision and client satisfaction as reported through client exit interviews. A study demonstrating impact on outcomes would require a more rigorous research design and a larger sample size, given the prevalence of maternal mortality. It would be unjustifiable to embark on a trial of that scale without first demonstrating feasibility and quality improvement. The most common causes of maternal mortality are related to delivery, including postpartum hemorrhage, and often cannot be predicted. However, there is ample literature (including World Health Organization guidelines) indicating that improving quality of ANC supports the prevention of common causes of maternal mortality, and this study was designed to improve the preventive functions that ANC care offers. [17] Additionally, there was a ceiling placed on satisfaction scores and therefore satisfaction scores remained high; thus, this study would have been better placed in facilities where there was general dissatisfaction with the quality of care at baseline.

The app was not designed to manage other aspects of clinic work, such as commodities management, staffing, and availability of key infrastructure. We note that implementing a decision support mobile application alone is not a universal fix, even when it is strongly associated with improvements in quality of ANC. Many other factors—such as adequate training, timely provision of commodities and drugs, and functioning equipment—are all necessary for high-quality ANC service. For example, even though proper guidance and a reminder were incorporated into the app about tetanus toxoid injection, we see a decline in tetanus toxoid coverage between baseline and endline because of the unavailability of the injections. Future projects should consider implementing mobile apps within a larger health system strengthening framework that includes additional training, supplies, and equipment in order to holistically address all aspects of ANC service quality.

The m4Change mobile ANC application was focused on improving quality of care through decision support protocols and client satisfaction. Quality of care scores for both technical and counseling domains, and the overall composite quality score showed a significant improvement as a result of the intervention. The overall client satisfaction with the ANC services also increased significantly from baseline to endline. At the time of publication, authors were unaware of other research looking at ANC and mobile applications impact on the quality of care so the authors are unable to draw comparisons with other research. More rigorous research is required to assess the app’s impact on health outcomes.
